# Reference tissue uptake of [18F]PSMA-1007 in positron emission tomography of recurrent prostate cancer

**DOI:** 10.1007/s00330-026-12496-6

**Published:** 2026-04-23

**Authors:** Bendik Skarre Abrahamsen, Ingerid Skjei Knudtsen, Andreas Julius Tulipan, Eivor Hernes, Trond Velde Bogsrud, Kirsten Margrete Selnæs, Håkon Johansen, Tone Frost Bathen, Mattijs Elschot

**Affiliations:** 1 Department of Circulation and Medical Imaging, Norwegian University of Science and Technology, Trondheim, Norway; 2 Nuclear Medicine/PET center, Department of Radiology, Haukeland University Hospital, Bergen, Norway; 3 Division of Radiology and Nuclear Medicine, Oslo University Hospital, Oslo, Norway; 4 PET Imaging Centre, University Hospital of North Norway, Tromsø, Norway; 5 PET-Centre, Aarhus University Hospital, Aarhus, Denmark; 6 Department of Radiology and Nuclear Medicine, Trondheim University Hospital—St. Olavs Hospital, Trondheim, Norway; 7 Research Department, Trondheim University Hospital—St. Olavs Hospital, Trondheim, Norway; 8 Faculty of Medicine and Health Sciences, Norwegian University of Science and Technology, Trondheim, Norway

**Keywords:** Prostatatic neoplasms, Prostate-specific membrane antigen, Positron-emission tomography, Reference standards, Spleen

## Abstract

**Introduction:**

Semi-quantitative uptake measurements are part of the PROMISE framework for PET/CT interpretation of tracers targeting the prostate-specific membrane antigen (PSMA), where tumor uptake is compared to uptake in reference tissues. [18F]PSMA-1007 has primarily hepatobiliary excretion and it is suggested to use spleen as reference tissue instead of liver. The aim of this study is to investigate the normal range and interpatient variability of [18F]PSMA-1007 uptake in reference tissue for prostate cancer patients, with particular focus on the splenic uptake.

**Materials and methods:**

A total of 102 [18F]PSMA-1007 PET/CT scans of prostate cancer patients with biochemical recurrence (BCR) from three hospitals were included in the analysis. SUV_max_ and SUV_mean_ measurements were performed in the blood pool, liver, spleen, and parotid glands. Results are reported as median with percentiles [5th, 95th].

**Results:**

For 15/102 (15%) and 20/102 (20%) of the patients, the SUV_max_ and SUV_mean_, respectively, were higher in the spleen than in the parotid glands. Median SUV_max_ of the reference tissues were 1.2 [0.6, 2.2] for blood, 15.8 [8.9, 26.2] for spleen, and 26.6 [14.5, 40.4] for the parotid glands. Median SUV_mean_ were 11.9 [6.6, 20.7] for spleen and 19.3 [11.2, 32.1] for the parotid glands.

**Conclusion:**

In this multicenter cohort of BCR patients, [18F]PSMA-1007 reference tissue uptake is reported in accordance with the PROMISE protocol. A substantial number of patients present with higher uptake in the spleen than in parotid glands, for reasons that are currently unexplained. Care should be taken when interpreting these values until further studies are conducted.

**Key Points:**

***Question**** The normal range for [18F]PSMA-1007 uptake values in PROMISE reference tissues are not well documented, challenging standardized lesion categorization in prostate cancer PET interpretation*.

***Findings**** In a multicenter cohort, splenic [18F]PSMA-1007 uptake exceeded parotid gland uptake in a notable subgroup (15%), leading to situations where PSMA expression scoring according to the PROMISE framework cannot be applied as intended*.

***Clinical relevance**** Unexpectedly high splenic uptake relative to parotid gland uptake of [18F]PSMA-1007 in some patients may affect lesion categorization; careful interpretation is advised*.

**Graphical Abstract:**

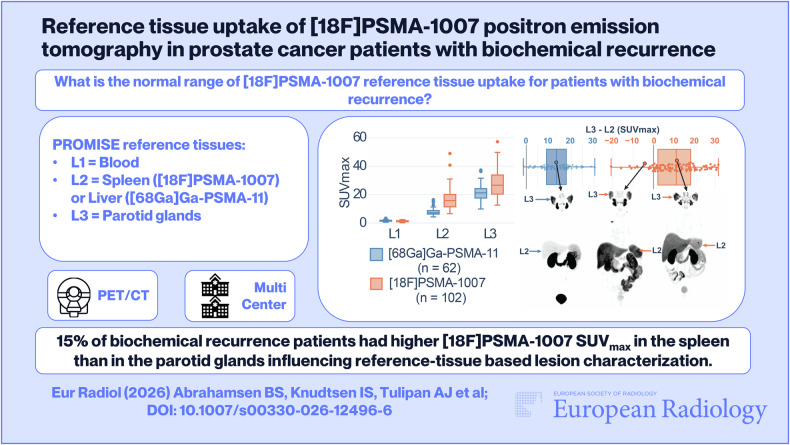

## Introduction

PET imaging with tracers targeting the prostate-specific membrane antigen (PSMA), PSMA-PET, has become an important diagnostic tool for patients with prostate cancer. Guidelines have been developed to standardize imaging procedures, reporting, interpretation, and response assessment [1–4]. Reflecting clinical practice, these include qualitative and semi-quantitative criteria for lesion scoring. The Prostate Cancer Molecular Imaging Standardized Evaluation Framework (PROMISE) aims to establish an extensive framework for interpretation and reporting suitable for both current and future response criteria [4, 5]. PROMISE-related parameters such as the molecular imaging TNM (miTNM) staging system—which classifies disease extent based on PSMA-PET findings—and the PSMA expression score—which grades lesion uptake relative to reference tissues [5], have been associated with outcome in several studies [6–11]. The PSMA expression score classifies lesion uptake relative to uptake in selected reference tissues, which vary depending on the PSMA-radioligand. The E-PSMA guideline, a consensus-based reporting standard, recommends both miTNM classification and the PSMA expression score, and also includes a 5-point lesion level reader’s confidence score [12].

Several PSMA-targeting radioligands are available. While [68Ga]Ga-PSMA-11 remains the most widely applied, [68Ga]Ga-PSMA-I&T, [18F]rhPSMA-7.3, [18F]DCFPyL, and [18F]PSMA-1007 are considered equivalent in terms of procedural and interpretative guidelines [5]. Based on differences in biodistribution, the tracers are categorized by their primary excretion pathway; urinary ([68Ga]Ga-PSMA-11, [68Ga]Ga-PSMA-I&T, [18F]DCFPyL, [18F]rhPSMA-7.3) or hepatobiliary ([18F]PSMA-1007). PROMISE recommends different reference tissues for each group: blood, liver, and parotid glands for urinary-excreted tracers, and blood, spleen, and parotid glands for hepatobiliary tracers [4].

[18F]PSMA-1007 is an established PSMA ligand [3] and widely applied in routine clinical practice. Few studies have addressed healthy tissue uptake, variability, and normal ranges for this tracer. Giesel et al [13] first described its biodistribution in a small cohort. More recently, Popescu et al [14] and Trädgårdh et al [15] reported uptake variability in normal tissues based on multicenter cohorts, but without linking findings to the PROMISE framework. Jansen et al [16] analyzed uptake for several prostate cancer tracers. Although [18F]PSMA-1007 was not included in the study, they emphasized the need to validate the spleen as a reference tissue for tracers with liver-dominant excretion due to high interpatient variability.

In this study, we aim to establish normal ranges and interpatient variability of reference tissue uptake in [18 F]PSMA-1007 PET in accordance with the PROMISE framework. We compare the uptake patterns to the more common [68Ga]Ga-PSMA-11 and evaluate the influence of imaging modality and post-injection time. Additionally, we examine the distribution of PSMA expression scores relative to the clinical interpretation of lesions.

## Methods

### Study design and ethical approval

This is a retrospective study based on data from two clinical studies approved by the Regional Committees for Medical and Health Research Ethics (REC). The first was retrospective, including prostate cancer patients with biochemical recurrence (BCR) referred for PSMA-PET at St. Olavs Hospital, Trondheim, Norway, between June 2018 and May 2019 (REC 10473). The second was prospective and multicenter (REC 83009, ClinicalTrials.gov ID NCT04298112), recruiting patients with BCR from May 2020 to April 2023. Signed informed consent was obtained for all patients in the prospective study; for the retrospective study, the REC waived the requirement for consent.

### Patient cohort

Inclusion criteria for the prospective study were: 1) biochemical relapse according to European Association of Urology (EAU) guidelines [17], and 2) eligibility for loco-regional pelvic salvage treatment. Exclusion criteria were (1) previous salvage therapy for recurrent prostate cancer, (2) contraindications for MRI (as PET/MRI was part of the protocol), (3) serious systemic disorders or cognitive impairment that could interfere with study participation or evaluation, (4) impaired renal function (eGFR < 30 mL/min/1.73 m^2^) due to contrast agent risk, and (5) androgen deprivation therapy (ADT) within the last three months. From the retrospective study, only patients who fulfilled these criteria were included in the present analysis. Patients were recruited from three centers: Center 1 (St. Olavs Hospital), Center 2 (Haukeland University Hospital, Bergen), and Center 3 (University Hospital of North Norway, Tromsø). PET images were acquired with [18F]PSMA-1007 or [68Ga]Ga-PSMA-11, depending on the center. The primary cohort (*n* = 164) includes all eligible PET/CT examinations from both studies. A secondary sub-cohort (*n* = 66) from Center 2 includes patients who underwent both PET/CT and PET/MRI on the same day following a single tracer injection, used to assess the effect of imaging modality and post-injection time.

### PET images

In the retrospective study, [18F]PSMA-1007 PET/CT was acquired 120 min post-injection. In the prospective study, in accordance with the study protocol, PET images were acquired at early time points (120 min for [18F]PSMA-1007; 50 min for [68Ga]Ga-PSMA-11) or late time points (180 min for [18F]PSMA-1007; 100 min for [68Ga]Ga-PSMA-11). At Center 2, all patients underwent both PET/CT and PET/MRI imaging on the same day following a single tracer injection. The imaging sequence was pseudo-randomized such that approximately half of the patients had PET/CT first and PET/MRI later, while the other half had PET/MRI first followed by PET/CT. Injected activities were: Center 1: 2.0 MBq/kg ([68Ga]Ga-PSMA-11) or 2.5 MBq/kg ([18F]PSMA-1007); Center 2: 300 MBq ([18F]PSMA-1007); Center 3: 150 MBq ([68Ga]Ga-PSMA-11). Reconstruction parameters are detailed in Table 1. Scatter correction methods varied by center and tracer: relative scatter scaling for [18F]PSMA-1007 and absolute scaling for [68Ga]Ga-PSMA-11 at Center 1; model-based relative scaling at Centers 2 and 3 [18, 19]

**Table 1:** Reconstruction parameters for PET/CT and PET/MR images

Reconstruction settings	PET/CT			PET/MR
Center	Center 1	Center 2	Center 3	Center 2
Scanner	Biograph mCT	Biograph Vision 600 Edge	Biograph Vision 600 Edge	Biograph mMR
Acquisition time	3 min/bed	1.1 mm/sec (FM)	0.7 mm/sec (FM)	5 min/bed (WB) 13 min/bed (pelvis)
Matrix size	256 × 256	440 × 440	440 × 440	344 × 344
Voxel size [mm]	3.18 × 3.18 × 3.00	1.65 × 1.65 × 3.00	1.65 × 1.65 × 3.00	2.09 × 2.09 × 2.03
Algorithm	OSEM 2i21s	OSEM 4i5s	OSEM 4i5s	OSEM 3i21s
Post-reconstruction filter	4 mm Gaussian	All pass	All pass	4 mm Gaussian
PSF	Yes	Yes	Yes	Yes
TOF	Yes	Yes	Yes	No (not available)

All scanners were Siemens systems (Siemens Healthineers)

*OSEM* ordered-subset expectation-maximization, *I* iterations, *s* subsets, *PSF* point-spread function modeling, *TOF* time-of-flight, *FM* flow motion, *WB* whole body

### Reference tissues measurements and readers

Reference tissue measurements of blood pool, liver, spleen, and parotid glands were performed by nuclear medicine physicians (E.H., A.J.T., H.J., and T.V.B.) [4]. SUV_max_ and SUV_mean_ were extracted according to predefined criteria outlined in Table 2. For the blood pool, only SUV_max_ was measured. The liver VOIs avoided large blood vessels, cysts, and other non-liver-tissue structures. The right parotid gland was used when the uptake was similar in both parotid glands, otherwise the parotid gland with the highest uptake was used [20].

**Table 2:** Reference tissues and predefined extraction criteria

Reference tissue	Size (diameter) and shape of ROI/VOI	Position
Blood pool	Circular	Aorta descendens
Liver	Spherical (2 cm)	Right lobe
Spleen	Spherical (2 cm) or organ segmentation^*^	Organ parenchyma
Parotid glands	Spherical 1 cm or organ segmentation^*^	Right gland

*ROI* region of interest, *VOI* volume of interest

^*^ Segmentation was performed using the autocontouring function in PET VCAR (AW-server, GE Healthcare) with estimated thresholding (gradient-based with a weight factor of 0.5 (PET VCAR User guide, 5914505-1EN Revision 1, 2022, p 66)

PSMA expression scores were as follows: 0 = below blood pool; 1 = above blood pool but below liver ([68Ga]Ga-PSMA-11) or spleen ([18F]PSMA-1007); 2 = above liver/spleen but below parotid gland; 3 = above parotid gland [4, 5]. Reference tissue uptake levels were denoted L1 (blood pool), L2 (liver or spleen), and L3 (parotid gland). All SUVs were normalized to body weight.

### Lesion analysis

Lesions were reported by experienced nuclear medicine physicians (> 5 years PSMA-PET experience). Each lesion was scored using a five-point scale analogous to the E-PSMA reader’s confidence score [12]: 1, benign; 2, probably benign; 3, equivocal; 4, probably malignant; and 5, malignant. Lesions scored > 3 were included in the analysis. PSMA expression scores were assigned to all lesions regardless of meeting the PROMISE criteria of having a morphologic correlate >10 mm in diameter [5].

### Statistical analysis

SUVs are reported as medians, with 5th and 95th percentiles to illustrate the typical range of values observed. The boxplots display the interquartile range as the box, with the median indicated by a horizontal line inside the box. Whiskers extend to the most extreme data points within 1.5 times the interquartile range from the first and third quartiles. Individual observations are displayed alongside each box. Statistical significance of differences in uptake levels was assessed using Wilcoxon signed rank test for paired samples and Mann–Whitney *U*-test for independent samples. Variance differences were assessed using the Brown–Forsythe test. Correlations between SUV and reference tissue uptake were evaluated using Spearman’s rank correlation. *p*-values < 0.05 were regarded as significant. Analyses were performed in Python using SciPy (v.1.10.1) [21] and pandas (v.2.0.3) [22].

## Results

### Reference tissue tracer uptake and variability

Patient characteristics are summarized in Table 3. Among those imaged with [18 F]PSMA-1007, 53 (51%) were scanned at the early time point, and 49 (48%) were scanned at the late time point; for [68Ga]Ga-PSMA-11, 21 (34%) patients were scanned early and 41 (66%) late. Reference tissues' uptake values are presented in Table 4 and Supplementary Tables 1-3. As expected, on the cohort level, median uptake increased monotonically from the blood pool (L1) to the liver/spleen (L2) and parotid glands (L3) for both tracers. [18F]PSMA-1007 showed significantly higher SUV_max_ in the blood pool than [68Ga]Ga-PSMA-11 (*p* < 0.001), with similar variance (*p* = 0.97).

**Table 3:** Overview of the patient cohort

Patient cohort	Center 1	Center 2	Center 3	Total
Number (18F/68Ga)	34/44	68/–	–/18	102/62
Age	69 [56, 79]	70 [59, 79]	67 [58, 75]	69 [56, 79]
Prior treatment				
Surgery	72	49	16	137
Radiotherapy	6	19	2	27
PSA level at imaging (ng/mL)	0.40 [0.15, 6.32]	0.50 [0.20, 6.75]	0.35 [0.15, 3.92]	0.40 [0.20, 6.18]
ISUP grade group (2/3/4/5)	14/33/24/7	0/28/39/1	0/7/9/2	14/68/72/10
Dose (MBq)				
[18F]PSMA-1007	216 [151, 288]	313 [282, 337]		300 [187, 335]
[68Ga]Ga-PSMA-11	152 [139, 161]		175 [149, 196]	153 [140, 186]
Weight (kg)	87 [71, 115]	83 [67, 106]	86 [73, 101]	85 [70, 113]

Age, PSA level, administered activity, and weight are given as median [5th percentile, 95th percentile]

**Table 4:** Reference tissue uptake measurements reported as median and 5th and 95th percentiles in brackets

Radionuclide	[18 F]PSMA-1007		[68Ga]Ga-PSMA-11	
Measurement tissue	SUV_max_	SUV_mean_	SUV_max_	SUV_mean_
Blood	1.2 [0.6, 2.2]	–	1.7 [0.7, 2.5]	–
Liver	16.8 [11.0, 23.7]	13.4 [8.3, 18.8]	7.2 [5.0, 12.6]	5.2 [3.1, 7.6]
Parotid glands	26.6 [14.5, 40.4]	19.3 [11.2, 32.1]	21.2 [11.5, 30.2]	13.7 [7.7, 18.6]
Spleen	15.8 [8.9, 26.2]	11.9 [6.6, 20.7]	9.7 [5.2, 17.0]	6.8 [3.2, 12.2]

Median uptake and interpatient variability were also significantly higher for [18F]PSMA-1007 at L2 and L3 (SUV_mean_ and SUV_max_, all *p* < 0.001). In addition, liver uptake showed broad interpatient variability, largely overlapping spleen uptake. Strong correlations between SUV_max_ and uptake level were observed for both tracers ([68Ga]Ga-PSMA-11: *r* = 0.94, [18F]PSMA-1007: *r* = 0.87). However, the uptake increase from L1 to L3 was not monotonic in all patients. For [18F]PSMA-1007, 15/102 (15%) patients had higher spleen than parotid gland SUV_max_, and 20/102 (20%) had higher SUV_mean_.

**Fig. 1:**
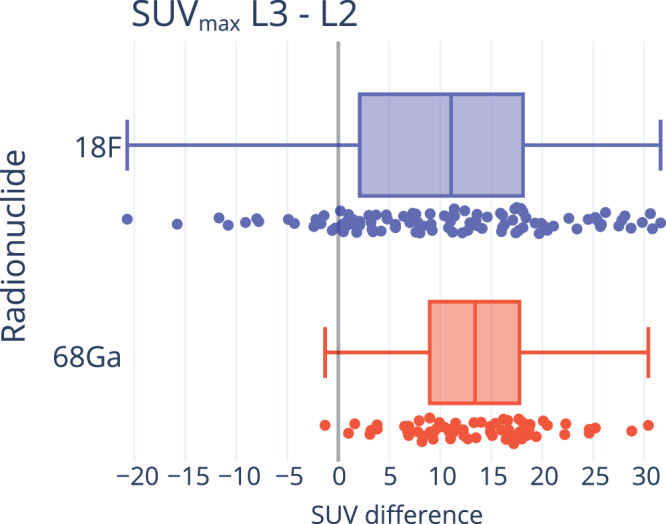
Horizontal box-plot and individual datapoints of the difference in SUV_max_ between the L3 reference level (parotid glands) and the L2 for the recommended reference tissue for each tracer. Spleen is used for [18F]PSMA-1007 and liver for [68Ga]Ga-PSMA-11

**Fig. 2:**
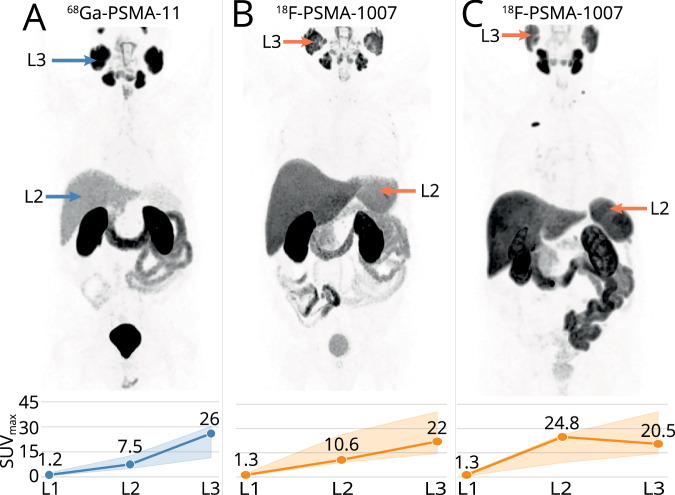
Maximum intensity projection images of three patients. **A** and **B** show [68Ga]Ga-PSMA-11 and [18F]PSMA-1007 images, respectively, with the difference between uptake level L3 and L2 close to the median value in their respective datasets. Panel **C** shows a patient for whom the uptake level L2 is higher than L3. Reference tissues for L2 and L3 are indicated with arrows. L1 is not annotated in the images due to the aorta not being clearly visible. SUV windows were chosen on a per-patient basis to yield good conspicuity and visual separation between reference tissue organs. An SUV window from 0 to 20 was used for **A** and **B**, and a window from 0 to 25 was used for panel **C**. The plots show the SUV_max_ for the corresponding patients, while the semi-transparent bands represent the 5th and 95th percentile values of the respective measurements for each tracer on the cohort level

The difference in SUV_max_ between the L3 and L2 uptake level for each tracer is shown in Fig. 1. For [68Ga]Ga-PSMA-11, only 1/62 (2%) of patients had higher uptake in the liver than the parotid glands for both SUV_max_ and SUV_mean_. Typical examples are provided in Fig. 2. Center-specific L2 > L3 rates are shown in Supplementary Table 4, and the distribution of L3–L2 differences showed similar qualitative patterns across centers (Supplementary Fig. 1). Neither primary treatment, PSA level at imaging, age, height, nor BMI had a clear association with atypical uptake patterns (Supplementary Fig. 2 and Table 5).

### Influence of post-injection time and modality

**Fig. 3:**
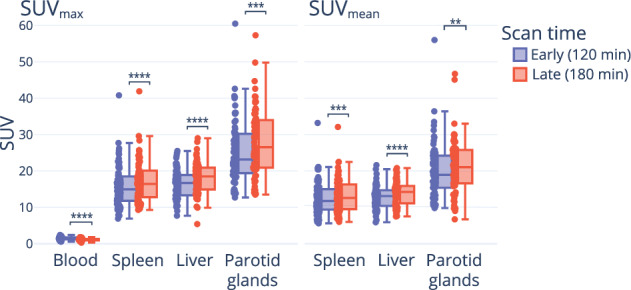
Reference tissue uptake with early and late acquisitions for [18F]PSMA-1007. The early acquisitions were acquired approximately 120 min post injection, and the late acquisitions approximately 180 min post injection. A slight but significant increase in SUV measurements is observed with the longer post injection time for all reference tissues except the blood pool (significant decrease). Differences were assessed using a Wilcoxon signed rank test, and significance levels are denoted as: ^*^(*p* < 0.05), ^**^(*p* < 0.01), ^***^(*p* < 0.001), ^****^(*p* < 0.0001)

Of 68 patients imaged at Center 2 with [18F]PSMA-1007 PET/CT at both early and late time points, two were excluded due to incomplete data, leaving 66 for analysis (Fig. 3). Among these, 29 were scanned early and 37 late. A significant decrease in blood pool SUV_max_ was observed at the late time point (*p* < 0.001), while all other reference tissues showed small but significant increases in SUV_max_ and SUV_mean_ (all *p* < 0.01). The largest absolute change was 3.4 in parotid gland SUV_max_; the largest relative change was a > 20% decrease in blood pool SUV_max_ from early to late uptake. On a cohort level, median uptake for L1, L2, and L3 remained well separated across time points. Of the 66 patients, 10 presented with L2 SUV_max_ higher than L3 SUV_max_ at the early time point. Six of these patients retained L2 SUV_max_ higher than L3 SUV_max_ at the late time point.

**Fig. 4:**
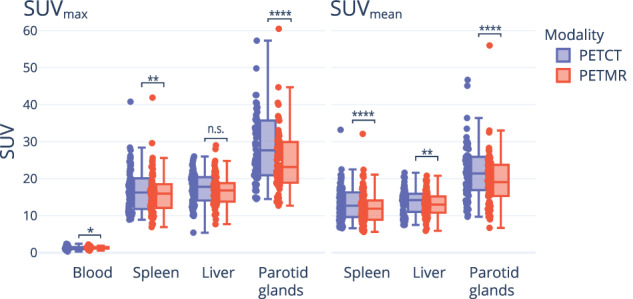
Comparison of healthy tissue uptake of [18F]PSMA-1007 between PET/CT and PET/MR, where slightly lower SUV measurements are observed for the latter in all reference tissues except blood, where a slight increase is observed, and liver SUV_max_, where no significant difference is found. Differences were assessed using a Wilcoxon signed rank test, and significance levels are denoted as: ^*^(*p* < 0.05), ^**^(*p* < 0.01), ^***^(*p* < 0.001), ^****^(*p* < 0.0001)

Comparison of [18F]PSMA-1007 PET/MR and PET/CT reference tissue uptake levels revealed minor modality-related differences (Fig. 4). PET/MR showed slightly lower SUV_max_ and SUV_mean_ in the spleen and parotid glands and a minor increase in blood SUV_max_. Liver SUV_mean_ was slightly lower on PET/MR, with no significant difference in SUV_max_. The largest absolute difference between PET/MR and PET/CT was 4.5 for the median SUV_max_ in the parotid glands. The largest relative difference was an 18.2% increase in blood pool SUV_max_ on PET/MR. On a cohort level, good separation is maintained between the median uptake levels L1, L2, and L3 for both modalities. Of the 66 patients, 9 patients had L2 SUV_max_ > L3 SUV_max_ on PET/MR, six of whom also showed this on PET/CT. A single patient presented with L2 SUV_max_ higher than L3 SUV_max_ only on PET/CT, and 3 patients only on PET/MR.

### Lesion analysis

**Fig. 5:**
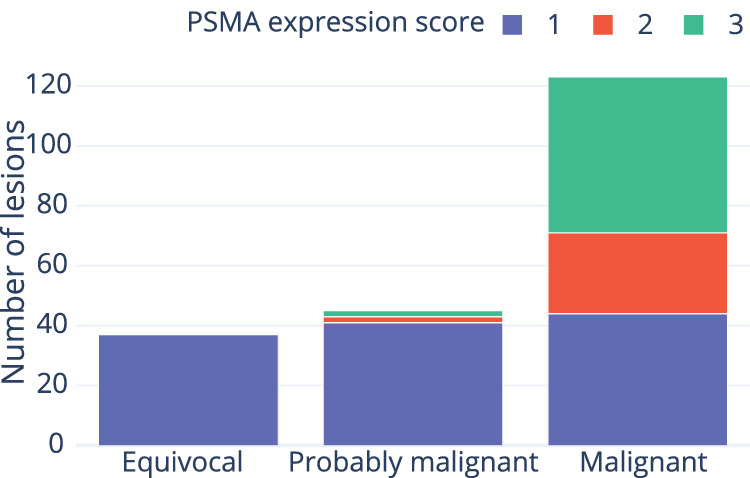
Uptake levels and E-PSMA reader’s confidence score for lesions recorded on [18 F]PSMA-1007 PET/CT. For [18F]PSMA-1007, a PSMA expression score of 1 is SUV_max_ higher than blood but lower than the spleen, 2 is SUV_max_ between the spleen and the parotid glands, and 3 is uptake above the parotid glands

In total, 205 lesions were scored as at least equivocal in the [18F]PSMA-1007 PET/CT: 37 equivocal, 45 probably malignant, and 123 malignant. Lesions considered equivocal, probably malignant, and malignant had median SUV_max_ of 5.3 [3.0, 13.1], 6.9 [6.9, 24.4], and 20.2 [8.1, 77.9], respectively. The distribution of lesions according to PSMA expression score and clinical score is shown in Fig. 5. All equivocal lesions, and all but four probably malignant lesions had a PSMA expression score of 1. Among malignant lesions, 52 lesions had a PSMA expression score of 3, 27 had a score of 2, and 44 had a score of 1.

## Discussion

We investigated [18F]PSMA-1007 in comparison to [68Ga]Ga-PSMA-11 uptake in reference tissues of patients with BCR after primary radical prostate cancer treatment. On a cohort level, median uptake increased from blood to spleen ([18F]PSMA-1007) or liver ([68Ga]Ga-PSMA-11) to the parotid glands. Notably, 15% of the [18F]PSMA-1007 patients had a spleen SUV_max_ exceeding the parotid glands, vs 2% of the [68G]Ga-PSMA-11 patients had a liver uptake higher than the parotid glands. For [18F]PSMA-1007, uptake rose with increasing time between tracer injection and image acquisition for all tissues except blood. Likewise, the SUVs in all reference tissues except blood were higher in PET/CT than in PET/MR images.

The monotonic increase in [18 F]PSMA-1007 uptake from blood to spleen to the parotid gland aligns with the initial evaluation by Giesel et al [13] and more recent findings by Popescu et al and Trädgårdh et al [14, 15], which also reported substantial interpatient variability in normal tissue uptake. However, neither related uptake to the PROMISE framework or PSMA expression score. Rahbar et al did not report parotid gland uptake [23], but observed large interpatient variability, again consistent with our findings. Similarly, variability was noted for tracers with primarily urinary excretion [16, 24], and intra-patient variability was demonstrated in a [18F]-DCFPyL test-retest-study [25]. Our observed decrease in blood pool uptake and increase in spleen, liver, and parotid gland for both [18F]PSMA-1007 and [68Ga]Ga-PSMA-11 with increasing time from injection to image acquisition is also in accordance with previous studies [13, 23, 24].

A major finding was the number of [18F]PSMA-1007 patients with spleen higher than parotid gland uptake, preventing assignment of a PSMA expression score of 2 per the PROMISE framework. On a cohort level, the spleen-parotid difference was smaller for [18F]PSMA-1007 than the liver-parotid difference for [68Ga]Ga-PSMA-11, resulting in a smaller difference between L2 and L3 for [18F]PSMA-1007, and consequently fewer lesions with a PSMA expression score of 2. Thus, lesion distribution across PSMA expression scores differs between the two tracers, and using the spleen as a reference organ for [18F]PSMA-1007 is not straightforward. Liver uptake for [18F]PSMA-1007 also showed substantial interpatient variability overlapping the range observed for spleen, suggesting that it does not provide a more stable alternative reference tissue.

Patients eligible for PSMA radioligand therapy must demonstrate PSMA-avid disease on imaging. Unlike our BCR cohort, these patients typically have advanced, castration-resistant disease, so our findings may not be directly transferable and should be explored in such cohorts. PROMISE recommends a PSMA expression score of 2 or 3 for PSMA radioligand therapy candidates. Clinical studies support that lesion uptake relative to reference tissues predicts treatment response, although different cutoffs have been used in retrospective analyses [8–10]. Both guidelines and clinical practice also consider additional factors such as the E-PSMA 5-point reader’s confidence score. In our study, over half the lesions classified as equivocal, probably malignant, and malignant had a PSMA expression score of 1 or 2. These findings highlight the need for caution when applying reference tissue thresholds in theranostic settings, as variability of normal tissue uptake—particularly in the spleen—may influence lesion scoring and patient selection. Within the PROMISE framework, PSMA expression level in general is to be evaluated visually, but it is also stated that quantitative analyses might be necessary [4]. Thus, a small difference in the spleen and parotid glands uptake level, visual or measured, will, in general, complicate the classification according to PSMA expression score.

The underlying reason why certain patients present with higher PSMA ligand uptake in the spleen than parotid glands remain unclear, but neither age, PSA level at imaging, height, weight or BMI seem to be relevant parameters (Supplementary Fig. 2). Additional analyses of demographic and clinical factors were performed (see Supplementary Tables 6 and 7), comparing tracer cohorts and testing for associations with atypical uptake patterns. None of the tested parameters showed a clear link to spleen > parotid gland uptake, even when significant differences between cohorts were present. ADT is known to affect PSMA expression and PSMA-PET imaging [26], but the patients included in the present analysis had no hormonal treatment for the last three months. Although the underlying biological mechanisms are not explored in this study, the results warrant further investigations into the biological mechanisms of [18F]PSMA-1007 uptake. For instance, differences in immune cell activity, splenic perfusion, or systemic inflammation could potentially contribute, but investigations into this would require access to additional clinical and laboratory data. These possible research avenues may help explain atypically high splenic uptake and could be explored in future mechanistic studies. However, such work is unlikely to influence the practical conclusion that the spleen is not a reliable reference organ for [18F]PSMA-1007 and, consequently, that the current PROMISE framework does not function as intended for a substantial subset of patients. Some degree of variability between centers is expected, as methodological differences can influence absolute SUV values. In the present data set, center-specific L2 > L3 rates were modestly different, but subgroup sizes, particularly at Center 2, were small and limit meaningful statistical comparisons. Importantly, the overall distribution of L3–L2 differences appeared qualitatively similar across centers (Supplementary Fig. 1), indicating that the atypical L2 > L3 uptake pattern reflects true interpatient variability rather than systematic center effects.

Comparing early and late acquisition times, the number of patients with L2 higher than L3 decreases with longer time after injection, although not sufficiently to explain the observation. As current clinical guidelines allow a broad time window between tracer injection and image acquisition, this is an important result.

Scanner and choice of reconstruction settings affect image quality and tracer quantification [27–31]. In this study, PET/CT acquisitions were performed on two systems (Siemens Biograph mCT and Siemens Biograph Vision 600 Edge) using site-specific clinical reconstruction protocols. The Vision system, a digital PET/CT, offers superior NEMA performance [32], though recovery coefficients are comparable [33]. For larger volumes, such as reference tissues, these differences are unlikely to affect SUV quantification substantially. PET/MR scans (Siemens Biograph mMR) included point spread function (PSF) correction, as time-of-flight (TOF) was unavailable. Øen et al [34] found similar contrast recovery and contrast-to-noise between mMR and mCT with PSF and TOF + PSF, respectively, suggesting that PET/MR and PET/CT differences are comparable to those between the two PET/CT systems. For patients scanned with both modalities (PET/CT and PET/MRI) after a single injection, the order of acquisition (PET/MR vs PET/CT) was balanced across patients. PSMA ligand uptake (SUV_max_ and SUV_mean_) was consistently higher on PET/CT than on PET/MR, but differences were small relative to interpatient variability and unlikely to affect the lesion PSMA expression scoring.

The patients included in this analysis were part of two clinical studies with standardized protocols for lesion reporting. For reference tissue measurements, the protocols recommend the use of circular or spherical volumes with defined diameters (Table 2), which were applied at Centers 2 and 3. At Center 1, measurements of the spleen and parotid glands were performed using the automatic segmentation function available in the clinical image analysis software system. Both the location and the size of the volume of interest can influence SUV quantification. In available literature, different methods and parameters have been applied or recommended to measure reference tissue uptake of PSMA tracers [4, 13, 16, 23, 24, 35]. Lawrence et al [35] evaluated [18F]DCFPyL PSMA PET/CT and PET/MR and found low inter-reader and inter-modality variability in SUV_mean_ for the liver, blood pool, and whole parotid gland. However, they reported substantially higher variability in the parotid gland when using small (1-cm) spherical ROIs, with differences driven by both ROI placement (superior, mid, inferior gland) and reader interpretation. In our data set, SUV_max_ and SUV_mean_ performed similarly in terms of stratifying reference tissue uptake levels. Moreover, the observed trends across early vs late image acquisitions and between imaging modalities were consistent for both parameters. This suggests that, despite methodological heterogeneity, our results are robust. Nevertheless, we acknowledge that SUV_mean_ is generally more sensitive to ROI definition, while SUV_max_ is more robust across varying measurement approaches.

Differences in administered tracer activities may also introduce variability in SUV measurements. However, all doses were within guideline-recommended ranges, and SUV calculations inherently normalize for injected activity. Inaccurate dose recording can affect SUV, but other parameters are far more influential sources of variability [36]. Thus, in large reference organs, minor differences in administered dose are unlikely to meaningfully impact SUV measurements.

To summarize, this study has some limitations, such as the use of different scanners and reconstruction protocols varying across centers. Multiple readers and differences in measurement methodology may also have introduced variability, although standardized protocols were applied. This also reflects clinical practice, and corresponding differences will be expected across hospitals employing the PROMISE framework and/or E-PSMA guidelines. As in most PSMA PET studies of BCR, histopathologic validation of lesions was not performed. This is rarely feasible in this setting due to the requirement for invasive biopsies, the multifocal distribution of disease, and the fact that such procedures may be considered unethical when not clinically indicated. Biopsies are generally reserved for cases where confirmation of metastatic disease is necessary for treatment planning and imaging findings are equivocal. According to current international guidelines, PSMA PET is the preferred imaging modality in the recurrence setting and does not require histopathologic verification [17]. Furthermore, the cohort consisted exclusively of patients with BCR after radical treatment, which may limit generalizability to other disease stages. Despite these limitations, this is, to our knowledge, the first study to systematically evaluate [18F]PSMA-1007 reference tissue uptake within the PROMISE framework. The multicenter design, harmonized lesion reporting, and consistent trends across imaging modality, uptake time, and SUV metrics support the robustness of our findings.

## Conclusion

For [18F]PSMA-1007, a subgroup of patients has higher PSMA ligand uptake in the spleen than the parotid glands, which affects categorization of lesions according to the PROMISE framework and PSMA expression score. The mechanisms behind this finding are unknown, and caution is warranted when interpreting these values. Further studies should explore this observation in other prostate cancer populations, especially patients with metastatic castration-resistant disease, to clarify its relevance for patient selection for PSMA radioligand therapy. Lesion classification according to PROMISE was otherwise robust with respect to imaging modality (PET/CT vs PET/MR), metrics (SUVmax vs SUVmean), and uptake time.

## Supplementary information

ELECTRONIC SUPPLEMENTARY MATERIAL
