# Supplementary material for: Reference tissue uptake of [18F]PSMA-1007 in positron emission tomography of recurrent prostate cancer
Source: Eur Radiol. 2026 Apr 23;36(8):6810–9. doi: 10.1007/s00330-026-12496-6 (PMC13342144; doi:10.1007/s00330-026-12496-6)
Supplement: Supplementary file 1 — ELECTRONIC SUPPLEMENTARY MATERIAL [file 330_2026_12496_MOESM1_ESM.pdf]

Reference tissue uptake of [18F]PSMA-1007 positron emission tomography in prostate cancer patients with biochemical recurrence
ELECTRONIC SUPPLEMENTARY MATERIAL

Supplementary material:

Reference tissue uptake of [18F]PSMA-1007 positron emission tomography in prostate cancer patients with biochemical recurrence.

Bendik Skarre Abrahamsen, Ingerid Skjei Knudtsen, Andreas Julius Tulipan, Eivor Hernes, Trond Bogsrud, Kirsten Margrete Selnæs, Håkon Johansen, Mattijs Elschot, Tone Frost Bathen

Supplementary table 1: Reference tissue measurements (SUV) for early vs late acquisition for [18F]PSMA-1007 PET/CT. The early acquisitions are acquired approximately 120 min post injection and the late acquisitions are acquired approximately 180 min post injection. The table contains 66 patients with the same-day PET/MR and PET/CT after a single tracer injection. Values are given as median [5th, 95th percentile].

Measurement	Tissue	Early	Late	*p*-value
Max	Blood pool	1.4 [0.8, 2.1]	1.1 [0.6, 1.7]	< 0.001
	Liver	16.7 [10.4, 23.2]	18.5 [12.2, 24.7]	< 0.001
	Parotid glands	23.2 [14.4, 40.2]	26.6 [14.7, 42.1]	< 0.001
	Spleen	15.0 [9.0, 25.4]	16.4 [10.0, 25.7]	< 0.001
Mean	Liver	13.1 [8.4, 17.8]	14.2 [8.6, 19.4]	< 0.001
	Parotid glands	19.0 [10.8, 32.2]	21.0 [9.9, 30.0]	< 0.001
	Spleen	11.8 [6.7, 20.7]	12.6 [7.0, 20.8]	< 0.001

Supplementary table 2: Reference tissue measurements (SUV) [18F]PSMA-1007 PET/CT and PET/MR. The table contains 66 patients with the same-day PET/MR and PET/CT after a single tracer injection. Values are given as median [5th, 95th percentile].

Measurement	Tissue	PET/CT	PET/MR	*p*-value
Max	Blood pool	1.1 [0.6, 1.9]	1.3 [0.8, 1.9]	0.045
	Liver	17.8 [10.6, 24.1]	16.8 [10.8, 24.2]	0.101
	Parotid glands	27.6 [16.7, 42.1]	23.2 [13.8, 39.2]	< 0.001
	Spleen	16.2 [9.9, 26.1]	16.0 [9.3, 24.8]	0.003
Mean	Liver	14.2 [8.4, 19.0]	13.0 [8.6, 18.7]	0.009
	Parotid glands	21.4 [11.8, 32.9]	19.0 [10.0, 28.2]	< 0.001
	Spleen	12.7 [7.2, 20.8]	11.9 [6.6, 20.7]	< 0.001

Supplementary table 3: Reference tissue measurements (SUV) for [18F]PSMA-1007 and [68Ga]Ga-PSMA-11 PET/CT for each center. Values are given as median [5th, 95th percentile].

Measurement	Tissue	Center	Max	Mean
18F	Blood pool	Center 1	1.3 [0.6, 2.2]	-
	Blood pool	Center 2	1.1 [0.6, 1.9]	-
	Liver	Center 1	14.4 [11.3, 23.0]	11.5 [8.3, 17.5]
	Liver	Center 2	17.5 [10.6, 24.0]	14.2 [8.4, 18.9]
	Parotid glands	Center 1	25.6 [13.4, 36.9]	16.2 [10.8, 26.7]
	Parotid glands	Center 1	26.9 [16.7, 41.9]	21.2 [11.9, 32.7]
	Spleen	Center 1	14.6 [7.1, 27.0]	10.1 [5.6, 18.3]
	Spleen	Center 2	16.4 [9.9, 26.0]	12.8 [7.2, 20.8]
68Ga	Blood pool	Center 1	1.4 [0.7, 2.0]	-
	Blood pool	Center 3	2.0 [1.4, 2.7]	-
	Liver	Center 1	7.3 [5.0, 9.8]	5.5 [3.4, 7.5]
	Liver	Center 3	7.1 [5.0, 15.6]	4.2 [2.7, 7.4]
	Parotid glands	Center 1	21.2 [11.6, 29,7]	12.4 [7.7, 16.8]
	Parotid glands	Center 3	21.2 [13.6, 31.3]	16.2 [11.1, 25.7]
	Spleen	Center 1	9,6 [5.2, 17.0]	6.4 [3.2, 12.1]
	Spleen	Center 2	9.9 [5.6, 17.6]	7.6 [3.1, 12.4]

Supplementary table 4: Number of patients imaged with [18F]PSMA-1007 L2>L3 stratified according to center.

Center	SUV_{max} L2 > L3	SUV_{mean} L2 > L3	Total
Center 1	8	10	78
Center 2	7	10	24

Supplementary table 5: Number of patients imaged with [18F]PSMA-1007 L2>L3 stratified according to prior treatment.

Prior treatment	SUV_{max} L2 > L3	SUV_{mean} L2 > L3	Total
Radical prostatectomy	11	15	78
Radiotherapy	4	5	24

Supplementary table 6: Continuous patient characteristics for patients scanned with [18F]PSMA-1007 and [68Ga]Ga-PSMA-11 PET/CT. Mean values are given with 95% bootstrap confidence intervals. Median values are given with the 5th and 95th percentile. Mann-Whitney U test was used to compute the p-values.

Measurement	Radionuclide	Mean	Median	p-value
Age (years)	[18F]PSMA-1007	68.9 [67.6, 70.2]	69 [56.1, 78.9]	0.475
	[68Ga]Ga-PSMA-11	67.9 [65.8, 69.6]	68.5 [56, 77.9]	
Weight (kg)	[18F]PSMA-1007	85.4 [82.9, 88.1]	83.5 [65.1, 106.9]	0.020
	[68Ga]Ga-PSMA-11	90.3 [87.6, 93.9]	86.5 [73.1, 113.5]	
Height (cm)	[18F]PSMA-1007	177.9 [176.6, 179.1]	178 [167.1, 189.0]	0.047
	[68Ga]Ga-PSMA-11	180.1 [178.6, 181.7]	180 [170, 190]	
PSA (ng/ml)	[18F]PSMA-1007	1.79 [1.36, 2.35]	0.41 [0.2, 7.1]	0.146
	[68Ga]Ga-PSMA-11	0.94 [0.64, 1.61]	0.40 [0.15, 3.80]	

Supplementary table 7: Categorical patient characteristics for patients scanned with [18F]PSMA-1007 and [68Ga]Ga-PSMA-11 PET/CT. Values are given as counts. Wilcoxon rank-sum test was used to compare ISUP grade groups between the populations and a Fisher's exact test was used to compare treatments in each population. RP = radical prostatectomy, RT = radiotherapy.

Measurement	Radionuclide	Counts	p-value
ISUP Grade group (2/3/4/5)	[18F]PSMA-1007	9/42/44/7	0.946
	[68Ga]Ga-PSMA-11	5/26/28/3	
Prior treatment (RP/RT)	[18F]PSMA-1007	78/24	0.002
	[68Ga]Ga-PSMA-11	59/3	

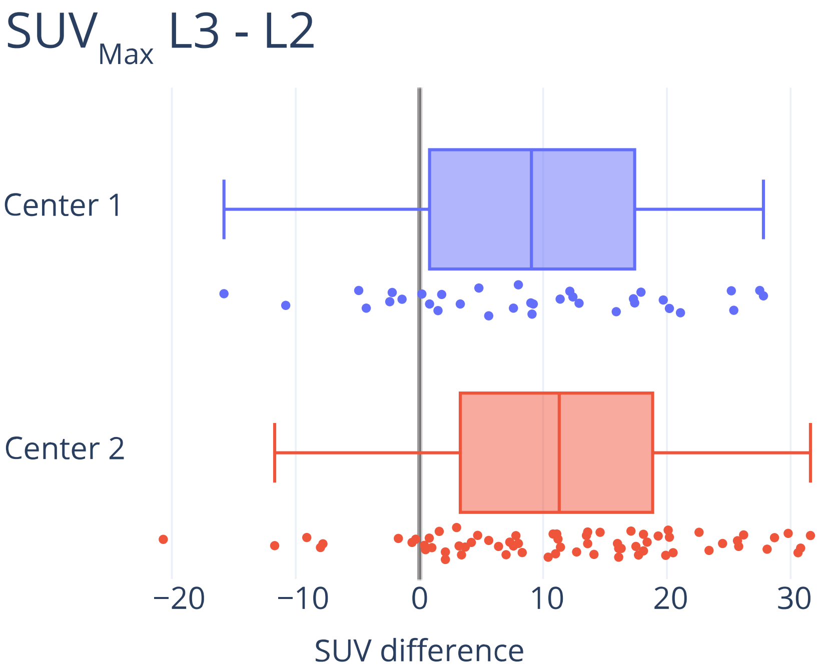

Supplementary figure 1: Horizontal box-plot and individual datapoints of the difference in SUV$_{max}$ between the L3 reference level (parotid glands) and the L2 (spleen) for the [18F]PSMA-1007 PET/CT data from each center . The box shows the interquartile range with median; whiskers extend to the most extreme values within 1.5 interquartile ranges from the first and third quartile

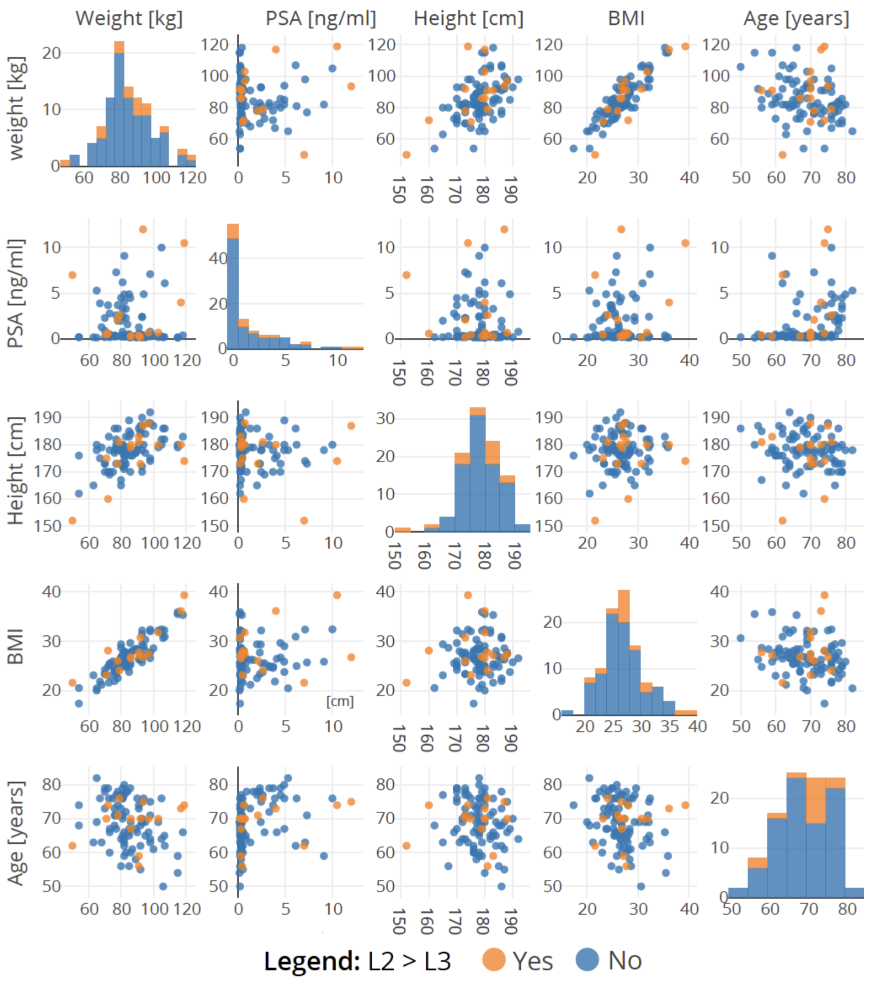

Supplementary figure 2: Scatter matrix showing selected clinical variables and whether the SUV_{max} uptake of the spleen (L2) was higher than the uptake level of the parotid glands (L3) for [18F]PSMA-1007. None of the selected clinical variables were able to predict the patients for which L2 > L3.
